# Discovery of 1-Pyrimidinyl-2-Aryl-4,6-Dihydropyrrolo [3,4-d]Imidazole-5(1*H*)-Carboxamide as a Novel JNK Inhibitor

**DOI:** 10.3390/ijms21051698

**Published:** 2020-03-02

**Authors:** Miyoung Jang, Youri Oh, Hyunwook Cho, Songyi Yang, Hyungwoo Moon, Daseul Im, Jung-Mi Hah

**Affiliations:** College of Pharmacy and Institute of Pharmaceutical Science and Technology, Hanyang University, Ansan 15588, Koreabestimda@hanyang.ac.kr (D.I.)

**Keywords:** JNK, RIPK, imidazole, Alzheimer’s disease, SAR

## Abstract

We designed and synthesized 1-pyrimidinyl-2-aryl-4, 6-dihydropyrrolo [3,4-d] imidazole-5(1*H*)-carboxamide derivatives as selective inhibitors of c-Jun-N-terminal Kinase 3 (JNK3), a target for the treatment of neurodegenerative diseases. Based on the compounds found in previous studies, a novel scaffold was designed to improve pharmacokinetic characters and activity, and compound **18a**, *(R)*-1-(2-((1-(cyclopropanecarbonyl)pyrrolidin-3-yl)amino)pyrimidin-4-yl)-2-(3,4-dichlorophenyl)-4,6-dihydro pyrrolo [3,4-d]imidazole-5(1*H*)-carboxamide, showed the highest IC_50_ value of 2.69 nM. Kinase profiling results also showed high selectivity for JNK3 among 38 kinases, having mild activity against JNK2, RIPK3, and GSK3β, which also known to involve in neuronal apoptosis.

## 1. Introduction

Alzheimer’s disease (AD) is one of the most common neurodegenerative diseases and features both amyloid β plaques and neurofibrillary tangles (NFTs) as pathological hallmarks [1]. Although the cause of AD, which affects many, is not clearly identified, many therapeutic agents have been studied to inhibit the formation of these two hallmarks, and we aim to develop a treatment for Alzheimer’s disease that targets and inhibits JNK3, which is deeply involved in the formation of amyloid β protein and NFT [2]. 

The c-Jun N-terminal kinase (JNK), also called stress-activated protein kinase, is a protein kinase that belongs to the mitogen-activated protein kinase (MAPK) family [3]. JNKs are encoded by the *jnk1*, *jnk2*, and *jnk3* genes [2], and there are approximately 10 isoforms with molecular weights of 46–55 kDa, depending on splicing. The signaling system of JNKs is activated by UV rays, inflammatory cytokines, and oxidative stress. The activated MKK4 and MKK7 phosphorylate the Thr183 and Tyr185 of JNKs. The activated JNKs promote the phosphorylation of proteins (AP-1, c-Jun, etc.) involved in cell apoptosis, proliferation, and differentiation [4,5,6,7,8]. JNK1 and JNK2 are expressed in most cells, whereas JNK3 is mainly expressed in the brain [9,10,11]. 

Most importantly, JNK3 expressed in the brain phosphorylates Thr668 of the amyloid precursor protein (APP), so that APP is located on the cell membrane and cleaved by β-secretase and γ-secretase to induce the formation of amyloid β protein [12,13]. The produced amyloid β protein is known to form amyloid plaques, causing neuronal cell apoptosis, and the amyloid β protein also causes positive feedback to reactivate JNK3 [12]. In addition, JNK3 phosphates Ser422 of the tau protein to form NFTs. The formed NFTs disrupt the transport of neurotransmitters by breaking down the structure of microfabrication in neurons, leading to the apoptosis of nerve cells [14].

We studied protein kinase inhibitors targeting JNK3 to develop effective treatments for Alzheimer’s disease by impeding these mechanisms.

## 2. Results and Discussion 

**Figure 1:**
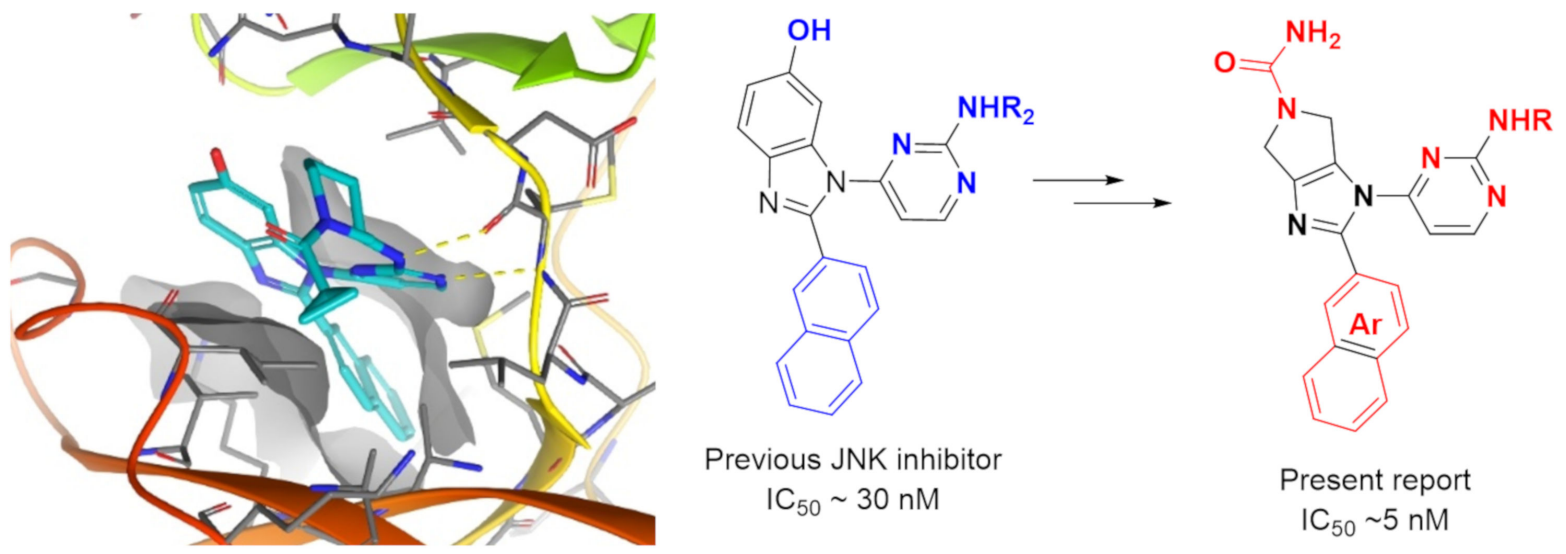
Docking structures of the previous c-Jun N-terminal kinase 3 (JNK3) inhibitor (PDB: 3OY1) and design of the present 1-pyrimidinyl-2-aryl-4, 6-dihydropyrrolo[3,4-d]imidazole-5(1*H*)-carboxamide scaffold. The important pharmacophores have been colored in blue in previous scaffold and red in present scaffold.

In previous studies [15], we discovered a 1-phenyl-2-pyrimidyl-1*H*-benzimidazole derivative, a hit compound that has JNK3 inhibitory activity, especially excellent selectivity versus other protein kinases. Therefore, we derivatized the hit compound further for better inhibitory activity on JNK3, and the lead compound, 1-(2-aminopyrimidin-4-yl)-2-(naphthalen-2-yl)-1*H*-benzo[d]imidazol-6-ol, was found. Moreover, we continued the efforts to improve the pharmacokinetic problem in the previous scaffold, and also to increase the activity against JNK3, having identified the main interactions through the docking study of the previous scaffold (hydrogen bonds in the hinge region, hydrophobic interaction of the aromatic ring, and the hydrogen bond of phenol in the benzimidazole scaffold). As a result, 1-pyrimidinyl-2-aryl-4, 6-dihydropyrrolo[3,4-d]imidazole-5(1*H*)-carboxamide derivatives were designed; these compounds had a five-membered ring, 2,5-dihydropyrrole, instead of the benzene in the previous benzimidazole scaffold (Figure 1).

**Scheme 1:**
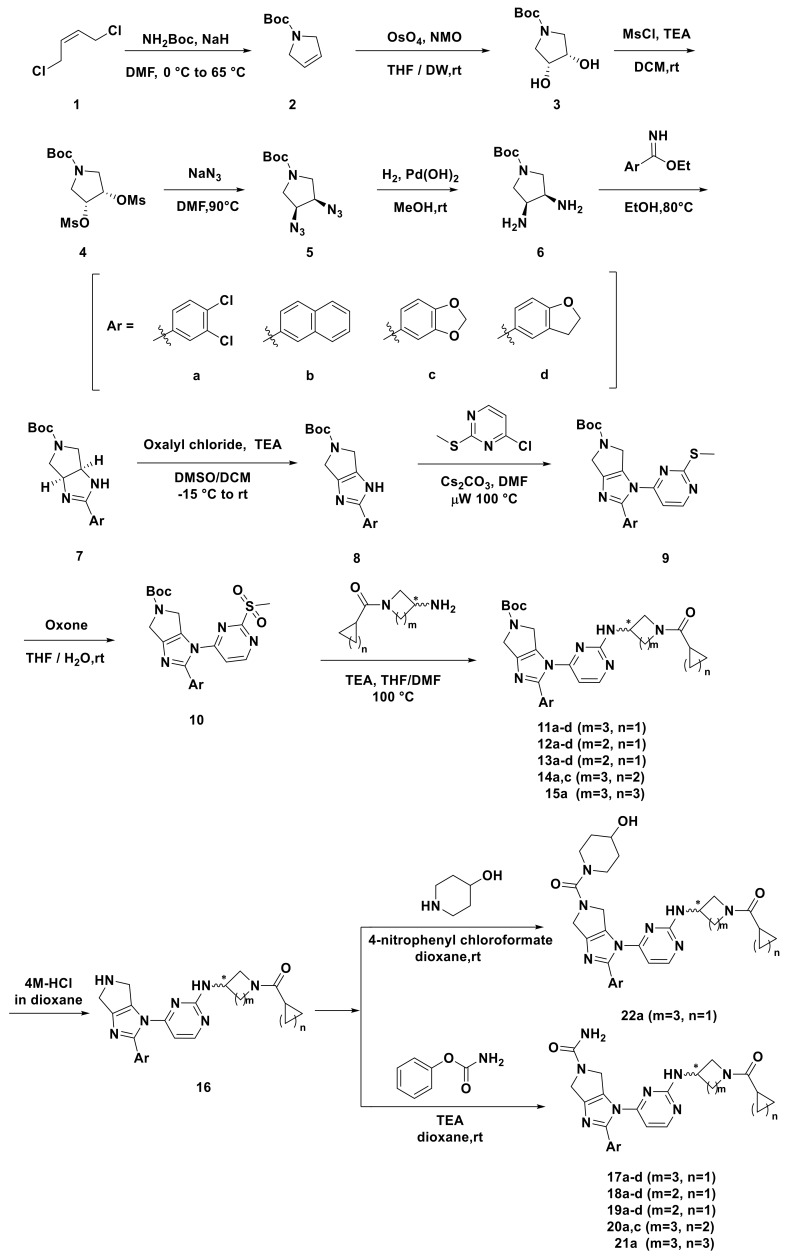
Synthesis of 1-pyrimidinyl-2-aryl-4,6-dihydropyrrolo[3,4-d]imidazole-5(1*H*)-carboxamide. (rt: room temperature, *: Stereocenter).

We proceeded with the synthesis of 1-pyrimidinyl-2-aryl-4, 6-dihydropyrrolo[3,4-d]imidazole-5(1*H*)-carboxamide derivatives via Scheme 1. For the synthesis of the core structure, dihydropyrrolo[3,4-d]imidazole-bicycle, we started to synthesize a 1,2-diaminopyrrolidine ring. The commercially available 1, 4-dichlorobut-2-ene **(1)** and *tert*-butyl carbamate were reacted to form Boc-pyrrolidine **(2)**. The dihydroxylation using osmium tetroxide was performed to give diol **(3)**, and it was transformed to diazide **(5)** through mesylation **(4) (**S_N_2 reaction). Following the reduction of diazide, using palladium as a catalyst gave the diamine **(6)**.

In order to form the next imidazoline **(7)** ring, the corresponding aryl imidate was reacted with diamino-*N*-Boc pyrrolidine; then, the *Swern oxidation* was accomplished to synthesize the 2,5-dihydropyrrolo imidazole core **(8)**. Next, 4-chloro-2-methylthio-pyrimidine was introduced to the core through S_N_Ar reaction under microwave irradiation **(9)**. The methyl sulfide was oxidized to methyl sulfone **(10)** by potassium peroximonosulfate and substituted with the amide-coupled amine group through another S_N_Ar **(11a–d, 12a–d, 13a–d, 14a, 14c and 15a).** The final products **(17a–d, 18a–d, 19a–d, 20a, 20c and 21a)** were obtained after Boc deprotection by HCl and phenylcarbamate treatment. Another final product **(22a)** was obtained using 4-nitrophenyl chloroformate.

After synthesis of all the compounds **(17a–d, 18a–d, 19a–d, 20a, 20c and 21a)**, the JNK3 inhibitory activity of each compound was evaluated (Table 1). Most of the synthesized compounds exhibited good activity against JNK3. In particular, **18a** showed the most potent activity against JNK3, with an IC_50_ value of 2.69 nM. Structure activity relationships (SARs) were inferred from potency data. First, when comparing the activity by the aryl group substitution, the compounds with the relatively large groups such as naphtyl and dichlorophenyl groups showed good inhibitory activity toward JNK3, rather than those with dioxolphenyl and dihydrobenzofuranphenyl groups **(a** and **b vs. c** and **d)**. We think that the aryl group occupied a larger hydrophobic space under the roof and induced hydrophobic interaction. This was assumed from the docking studies of the previous inhibitor of JNK3. Moreover, the napthyl and dichlorophenyl rings have higher electron densities, so could form stronger interactions with the surrounded residues, supporting better activities. Secondly, when the piperidin-4-ol **(17a)** was substituted in the position of the carboxamide in 2, 5-dihydropyrrolo-1-carboxamide, the activity falls to half that of the corresponding carboxamide **(17a vs. 22a)**. Next, when the cyclopropyl group in the solvent exposure part was replaced with a cyclobutyl or cyclopentyl group, the inhibitory activity decreased approximately two- to three-fold **(17a vs. 20a, 20c,** and **21a)**. In an effort to reduce the molecular weight, the piperidine ring was diversified into pyrrolidine with less carbon atoms (*n* = 2). Surprisingly, when *(R)*-aminopyrrolidine was coupled to the pyrimidyl group instead of the *(S)*-aminopiperidine, the activities were increased by approximately seven- to ten-fold **(17 vs. 19)**. Interestingly, when *(R)*-aminopyrrolidine was introduced, the activity was significantly increased by approximately four- to five-fold **(17 vs. 18)**. This also suggested that the size and configuration of the amino group in the ring should be considered important for binding, even in the solvent exposure part for optimal extra-hydrogen bonding. The extra hydrogen bonding seemed more plausible in *(R)*-pyrrolidine **(18)** than in the cases of *(S)*-piperidine **(17)** and *(S)*-pyrrolidine **(19)**.

**Figure 2:**
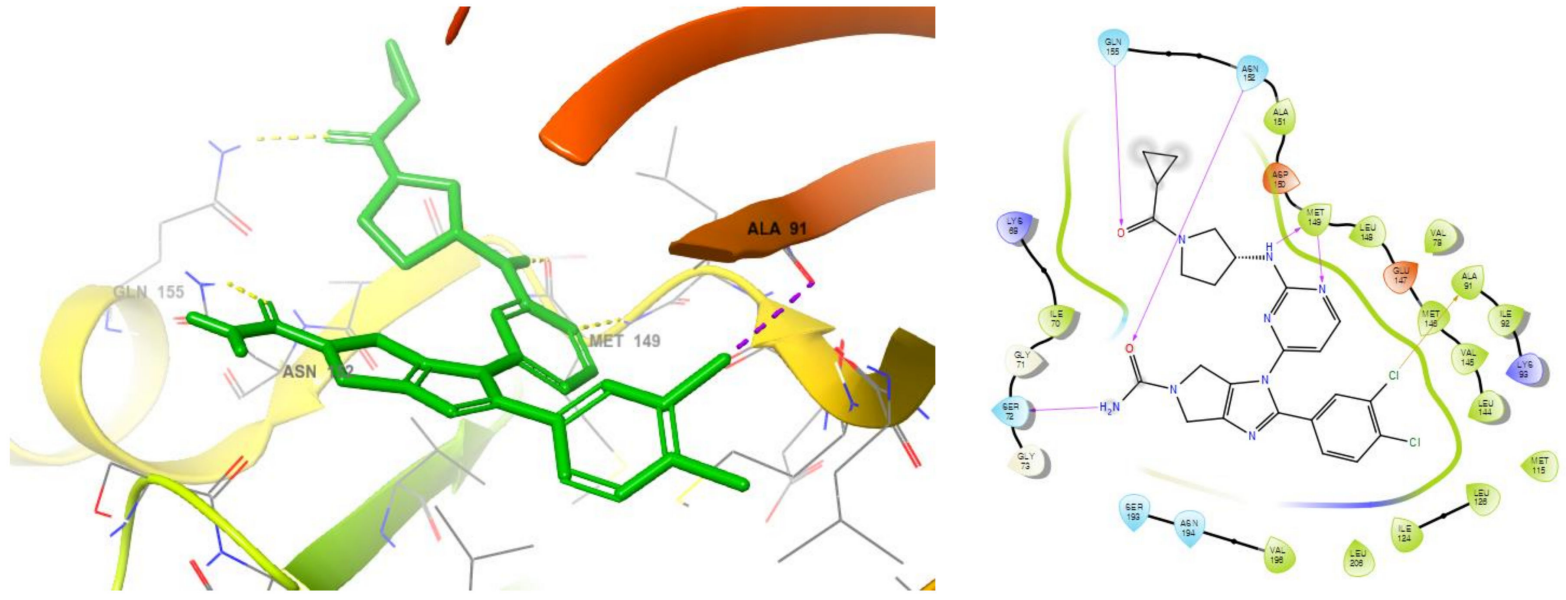
Docking structures of **18a** at JNK3 (PDB: 3OY1) and 2D interaction map.

A docking study was conducted to understand the binding mode of the novel JNK3 inhibitor **18a** (Figure 2). When we performed the docking experiment of **18a** with a known JNK3 structure (3OY1), it was shown that many of the interactions could contribute to complex stabilization. First, the amino pyrimidine is a hinge binder and forms two hydrogen interactions with the Met149 of JNK3. The oxygen of cyclopropyl carboxamide in compound **18a** could form hydrogen bond interaction with Gln155 in the extended hinge region. The two hydrogen bonds were monitored between the oxygen in the carboxamide of 2,5-dihydropyrrole and the side chain of Asn152, and between the NH_2_ in the carboxamide of 2,5-dihydropyrrole-1-carboxamide and the side chain of Ser72. Finally, the dichlorophenyl ring of compound **18a** fits into the hydrophobic pocket and forms a halogen bond with Ala93.

Next, we performed kinases panel screening in duplicate for compound **18a** on 38 different protein kinases at a single-dose concentration of 10μM (Table 2). The compound **18a** was indeed a selective JNK3 inhibitor with an excellent selectivity profile, only having slight activities on JNK2, GSK3β, and RIPK3 more than 50%. When we further determine the IC_50_ of **18a** on these three protein kinases and compared with it on JNK3, the selectivity was still maintained. And since the GSK3β and RIPK3 are said to be associated with neurodegenerative disease caused by all neuronal apoptosis [18,19,20,21,22,23], we could manipulate these characters of **18a** for further developments.

## 3. Materials and Methods

### 3.1. Chemistry

#### 3.1.1. General Chemical Methods

All chemicals were of reagent grade and were purchased from Sigma-Aldrich, Inc. (Seoul, Korea). Separation of the compounds by column chromatography was carried out with silica gel 60 (200–300 mesh ASTM, E. Merck, Darmstadt, Germany). The quantity of silica gel used was 50–100 times the weight charged on the column. Thin layer chromatography (TLC) was run on silica gel-coated aluminum sheets (silica gel 60 GF254, E. Merck, Darmstadt, Germany) and visualized under ultraviolet (UV) light (254 nm). Both ^1^H Nuclear Magnetic Resonance (NMR )and ^13^C NMR spectra were recorded on a Bruker model digital AVANCE III 400 MHz spectrometer (Billerica, MA, USA) at 25 °C using tetramethylsilane (TMS) as an internal standard. High-resolution Mass Spectra (HR/MS) experiments were conducted with a Finnigan LTQ Orbitrap mass spectrometer (Thermo Fisher Scientific Inc., New York, NY, USA) operated in positive-ion electrospray mode.

#### 3.1.2. General Syntheses of tert-Butyl 2, 5-Dihydro-1H-Pyrrole-1-Carboxylate (2)

Compound **1** (4.65 mmol) was dissolved in dimethyl formamide (5 mL), and then sodium hydride (10.23 mmol) was added at 0 °C and stirred for 10 min. Compound **1** was added to the mixture and stirred at room temperature for 15 min, followed by stirring at 65 °C for 4 to 6 h. It was then cooled to ambient temperature, extracted with an organic layer (Ethylacetate:n-Hexane (EA:HEX) = 1:4), and washed with water. This was followed by drying with anhydrous magnesium sulfate and evaporation of the solvent to obtain compound **3** as a yellow oil. (45%); ^1^H NMR (400 MHz, CDCl_3_): δ 5.76 (s, 2H), 4.11 (s, 4H), 1.47 (s, 9H); HRMS m/z calcd for C9H15NO2 169.2240; found 170.0130 (M+H+).

#### 3.1.3. General Syntheses of Tert-Butyl (3R, 4S)-3, 4-Dihydroxypyrrolidine-1-Carboxylate (3)

Compound **2** (6.3 mmol) was dissolved in tetrahydrofuran (15.8 mL) and we then slowly dropped the mixture of osmium tetroxide (0.113 mmol) and N-methylmorpholine-N-oxide (8.29 mmol) in 15.8 mL of water. The mixture was stirred at room temperature for 3 to 5 h. It was then concentrated in vacuo, extracted with ethyl acetate, and washed with water. This was followed by drying with anhydrous magnesium sulfate, evaporation of the solvent, and then purification of the product by column chromatography on silica gel using a mobile phase of EA: HEX (3: 1) to obtain compound **3** as a yellow oil. (59%); ^1^H NMR (400 MHz, CDCl_3_): δ 4.25 (qd, J = 4.4, 2.1 Hz, 2H), 3.59 (dd, J = 11.4, 5.7 Hz, 2H), 3.34 (dd, J = 11.3, 3.8 Hz, 2H), 1.45 (s, 9H); HRMS m/z calcd for C9H17NO4 203.2380; found 204.4411 (M+H+).

#### 3.1.4. General Syntheses of Tert-Butyl (3R, 4S)-3, 4-Bis((Methylsulfonyl)Oxy)Pyrrolidine-1-Carboxylate (4)

Compound **3** (0.96 mmol) was dissolved in dichloromethane (4.8 mL), and then methanesulfonyl chloride (2.11 mmol) and triethylamine (2.11 mmol) were added and stirred at room temperature for 30 min to 1 h. The reaction mixture was washed with water and brine. This was followed by drying with anhydrous magnesium sulfate and evaporation of the solvent to obtain compound **4** as a white solid. (90%); ^1^H NMR (400 MHz, CDCl_3_): δ 5.17 (t, J = 4.0 Hz, 2H), 3.84–3.74 (m, 2H), 3.68–3.59 (m, 2H), 3.14 (d, J = 5.6 Hz, 6H), 1.46 (s, 9H); HRMS m/z calcd for C11H21NO8S2 359.4080; found 360.0501 (M+H+).

#### 3.1.5. General Syntheses of Tert-Butyl (3S, 4R)-3,4-Diazidopyrrolidine-1-Carboxylate (5)

Compound **4** (3.3 mmol) was dissolved in dimethylformamide (33 mL), and then sodium azide (33 mmol) was added and the mixture was stirred at 90 °C for 24 h. The mixture was then cooled to ambient temperature, extracted with ethyl acetate, and washed with brine. After drying with anhydrous magnesium sulfate and concentration of the solvent in vacuo, the product was purified by column chromatography on silica gel using a mobile phase of EA:HEX (1:4) to obtain compound **5** as a yellow oil. (79%); ^1^H NMR (400 MHz, CDCl_3_): δ 4.08 (d, J = 3.3 Hz, 2H), 3.63 (dd, J = 8.8, 5.5 Hz, 2H), 3.44 (ddd, J = 16.3, 10.8, 4.1 Hz, 2H), 1.46 (s, 9H); HRMS m/z calcd for C9H15N7O2 253.2660; found 254.3558 (M+H+).

#### 3.1.6. General Synthesis of Tert-Butyl (3S, 4R)-3,4-Diaminopyrrolidine-1-Carboxylate (6)

Compound **5** (2.6 mmol) was dissolved in methanol (10.4 mL), and then palladium hydroxide on carbon (0.52 mmol) was added. It was stirred for 4 h at room temperature under hydrogen gas. The mixture was filtered through a celitepad, and the filtrate was concentrated to obtain compound **6** as a yellow oil. (98%); ^1^H NMR (400 MHz, DMSO-d_6_): δ 3.26 (dd, J = 10.9, 5.9 Hz, 3H), 3.14 (dq, J = 9.6, 4.8 Hz, 2H), 2.95 (dd, J = 10.6, 4.9 Hz, 2H), 1.38 (s, 9H). HRMS m/z calcd for C9H19N3O2 201.2700; found 202.3284 (M+H+).

#### 3.1.7. General Syntheses of Compounds 7a-d

Tert-Butyl (3aS,6aR)-2-(3,4-Dichlorophenyl)-3a,4,6,6a-Tetrahydropyrrolo-Imidazole-5(1H)-Carboxylate **(7a)**

Compound **6** (2.69 mmol) and aryl-substituted imidate (2.5 mmol) were dissolved in ethanol (13.4 mL) and stirred at 80 °C for 1 to 2 h. The mixture was cooled to ambient temperature and then concentrated in vacuo. The concentrated mixture was extracted with ethyl acetate and washed with brine. This was followed by drying with anhydrous magnesium sulfate, and the solvent was purified by column chromatography on silica gel using a mobile phase of EA:HEX (3:1) to obtain compound **7a** as a white solid. (47%); ^1^H NMR (400 MHz, MeOD): δ 7.96 (d, J = 2.0 Hz, 1H), 7.71 (dd, J = 8.4, 2.0 Hz, 1H), 7.63 (d, J = 8.4 Hz, 1H), 4.65 (s, 2H), 3.71 (d, J = 12.0 Hz, 2H), 3.55–3.44 (m, 2H), 1.43 (s, 9H); HRMS m/z calcd for C16H19Cl2N3O2 356.2470; found 357.4684 (M+H+).

*Tert*-Butyl *(3aS,6aR)*-2-(Naphthalen-2-yl)-3a,4,6,6a-Tetrahydropyrrolo[3,4-d]Imidazole-5(1H)-Carboxylate **(7b)**

Compound **7b** was obtained as a white solid by the same procedure as above. (crude yield: 95%); ^1^H NMR (400 MHz, DMSO-d_6_): δ 8.77 (d, J = 1.4 Hz, 1H), 8.18 (d, J = 8.7 Hz, 1H), 8.10–8.03 (m, 3H), 7.74 (ddd, J = 14.4, 7.9, 1.2 Hz, 2H), 4.94 (d, J = 1.4 Hz, 2H), 3.82 (d, J = 12.5 Hz, 2H), 3.45 (d, J = 12.4 Hz, 2H), 3.32 (s, 1H), 1.32 (s, 9H); HRMS m/z calcd for C20H23N3O2 337.4230; found 338.3165 (M+H+).

*Tert*-Butyl *(3aS,6aR)*-2-(Benzo[d][1,3]Dioxol-5-yl)-3a,4,6,6a-Tetrahydro Pyrrolo[3,4-d]Imidazole-5(1*H*)-Carboxylate **(7c)**

Compound **7c** was obtained as a white solid by the same procedure as above. (crude yield: 98%); ^1^H NMR (400 MHz, DMSO-d_6_): δ 7.68 (d, J = 11.4 Hz, 2H), 7.21 (d, J = 8.1 Hz, 1H), 6.21 (s, 2H), 4.85 (s, 2H), 3.76 (d, J = 12.5 Hz, 2H), 3.39 (d, J = 12.1 Hz, 2H), 3.32 (s, 1H), 1.34 (s, 9H); HRMS m/z calcd for C17H21N3O4 331.3720; found 332.3018 (M+H+).

*Tert*-Butyl *(3aS,6aR)*-2-(2,3-Dihydrobenzofuran-5-yl)-3a,4,6,6a-Tetrahyd Ropyrrolo[3,4-d]Imidazole-5(1*H*)-Carboxylate **(7d)**

Compound **7d** was obtained as a white solid by the same procedure as above. (crude yield: 98%); ^1^H NMR (400 MHz, CDCl_3_): δ 8.15 (s, 1H), 8.08 (d, J = 8.5 Hz, 1H), 6.74 (d, J = 8.5 Hz, 1H), 4.85 (s, 2H), 4.62 (t, J = 8.8 Hz, 2H), 4.01 (d, J = 12.5 Hz, 2H), 3.44 (d, J = 12.1 Hz, 2H), 3.18 (t, J = 8.7 Hz, 2H), 1.39 (s, 9H); HRMS m/z calcd for C18H23N3O3 329.4000; found 330.3653 (M+H+).

#### 3.1.8. General Syntheses of Compounds 8a-d

*Tert*-Butyl 2-(3,4-Dichlorophenyl)-4,6-Dihydropyrrolo[3,4-d]Imidazole-5 (1*H*)-Carboxylate **(8a)**

Oxalyl chloride (0.59 mmol) and dimethyl sulfoxide (1.18 mmol) were dissolved in 7 mL of dichloromethane, stirred at −78 °C for 10 min, and then slowly added to compound 7a (0.59 mmol) dissolved in 5 mL of dichloromethane at −78 °C for 30 min. Then, triethylamine (5.9 mmol) was added slowly and stirred at room temperature for 1 h 30 min. The reaction mixture was washed with water and brine, followed by drying with anhydrous magnesium sulfate and concentration of the solvent in vacuo to obtain compound **8a** as a white solid. (50%); ^1^H NMR (400 MHz, Dimethyl sulfoxide-d_6_ (DMSO-d_6_): δ 12.82 (d, J = 24.4 Hz, 1H), 8.41 (s, 1H), 8.07 (d, J = 8.6 Hz, 1H), 7.99–7.88 (m, 3H), 7.59–7.48 (m, 2H), 4.50 (s, 2H), 4.33 (d, J = 10.1 Hz, 2H), 1.47 (s, 9H); HRMS m/z calcd for C16H17Cl2N3O2 354.2310; found 355.4510 (M+H+).

*Tert*-Butyl 2-(Naphthalen-2-yl)-4,6-Dihydropyrrolo[3,4-d]Imidazole-5(1*H*)-Carboxylate **(8b)**

Compound **8b** was obtained as a white solid by the same procedure as above. (51%); ^1^H NMR (400 MHz, DMSO): δ 12.82 (d, J = 24.4 Hz, 1H), 8.41 (s, 1H), 8.07 (d, J = 8.6 Hz, 1H), 7.99–7.88 (m, 3H), 7.59–7.48 (m, 2H), 4.50 (s, 2H), 4.33 (d, J = 10.1 Hz, 2H), 1.47 (s, 9H); HRMS m/z calcd for C20H21N3O2 335.4070; found 336.2996 (M+H+).

*Tert*-Butyl 2-(Benzo[d][1,3]Dioxol-5-yl)-4,6-Dihydropyrrolo[3,4-d]Imidazole-5(1*H*)-Carboxylate **(8c)**

Compound **8c** was obtained as a white solid by the same procedure as above. (50%); ^1^H NMR (400 MHz, CDCl_3_): δ 7.32 (dd, J = 10.6, 1.6 Hz, 2H), 6.81 (d, J = 8.0 Hz, 1H), 5.99 (s, 2H), 4.49–4.39 (m, 4H), 1.51 (s, 9H); HRMS m/z calcd for C17H19N3O4 329.3560; found 330.3209 (M+H+).

*Tert*-Butyl 2-(2,3-Dihydrobenzofuran-5-yl)-4,6-Dihydropyrrolo[3,4-d]Imidazole-5(1*H*)-Carboxylate **(8d)**

Compound **8d** was obtained as a white solid by the same procedure as above. (50%); ^1^H NMR (400 MHz, CDCl_3_): δ 7.75 (s, 1H), 7.55 (dd, J = 8.3, 1.9 Hz, 1H), 6.78 (d, J = 8.3 Hz, 1H), 4.61 (t, J = 8.8 Hz, 2H), 4.50–4.41 (m, 4H), 3.22 (t, J = 8.7 Hz, 2H), 1.51 (s, 9H); HRMS m/z calcd for C18H21N3O3 327.3840; found 328.3040 (M+H+).

#### 3.1.9. General Syntheses of Compounds 9a–d

*Tert*-Butyl 2-(3,4-Dichlorophenyl)-1-(2-(Methylthio)Pyrimidin-4-yl)-4,6-Dihydropyrrolo[3,4-d]Imidazole-5(1*H*)-Carboxylate **(9a)**

Compound **8a** (0.75 mol), 4-chloro-2-(methylthio)pyrimidine (0.75 mmol) and cesium carbonate (0.9 mmol) were dissolved in 7.5 mL of N, N-dimethylformamide and stirred for 2 h at 100 °C in a microwave reactor. After 2 h, the reaction mixture was extracted with ethyl acetate and washed with water and brine. After drying over anhydrous magnesium sulfate and concentration, the product was purified by column chromatography on silica gel using a mobile phase of EA:HEX (1:2) to obtain compound **9a** as a yellow solid. (50%); ^1^H NMR (400 MHz, CDCl_3_) δ 8.28 (dd, J = 7.6, 5.5 Hz, 1H), 8.13 (d, J = 16.5 Hz, 1H), 7.88 (t, J = 8.7 Hz, 3H), 7.62–7.53 (m, 3H), 6.42 (d, J = 5.5 Hz, 1H), 4.86–4.79 (m, 2H), 4.59 (s, 2H), 2.55 (d, J = 3.4 Hz, 3H), 1.54 (s, 9H).; HRMS m/z calcd for C21H21Cl2N5O2S 477.3920, Found 478.4457 (M+H+).

*Tert*-Butyl 1-(2-(Methylthio)Pyrimidin-4-yl)-2-(Naphthalen-2-yl)-4,6-Dihydropyrrolo [3,4-d]Imidazole-5(1*H*)-Carboxylate **(9b)**

Compound **9b** was obtained as a yellow solid by the same procedure as above. (22%); ^1^H NMR (400 MHz, CDCl3): δ 8.28 (dd, J = 7.6, 5.5 Hz, 1H), 8.13 (d, J = 16.5 Hz, 1H), 7.89 (d, J = 9.0 Hz, 3H), 7.62–7.53 (m, 2H), 7.53–7.47 (m, 1H), 6.42 (d, J = 5.5 Hz, 1H), 4.86–4.79 (m, 2H), 4.59 (s, 2H), 2.55 (d, J = 3.4 Hz, 3H), 1.54 (s, 9H); HRMS m/z calcd for C25H25N5O2S 459.5680; found 460.4038 (M+H).

*Tert*-Butyl 2-(Benzo[d][1,3]Dioxol-5-yl)-1-(2-(Methylthio)Pyrimidin-4-yl)-4,6-Dihydropyrrolo[3,4-d]Imidazole-5(1*H*)-Carboxylate **(9c)**

Compound **9c** was obtained as a yellow solid by the same procedure as above. (47%); ^1^H NMR (400 MHz, CDCl3): δ 8.35 (dd, J = 5.5, 1.1 Hz, 1H), 7.00–6.93 (m, 2H), 6.85 (d, J = 7.9 Hz, 1H), 6.46 (t, J = 5.6 Hz, 1H), 6.04 (s, 2H), 4.83–4.72 (m, 2H), 4.54–4.45 (m, 2H), 2.56 (d, J = 2.1 Hz, 3H), 1.52 (d, J = 1.8 Hz, 9H); HRMS m/z calcd for C22H23N5O4S 453.5170; found 454.3178 (M+H+).

*Tert*-Butyl 2-(2,3-Dihydrobenzofuran-5-yl)-1-(2-(Methylthio)Pyrimidin-4-yl)-4,6-Dihydropyrrolo[3,4-d]Imidazole-5(1*H*)-Carboxylate **(9d)**

Compound **9d** was obtained as a yellow solid by the same procedure as above. (52%); ^1^H NMR (400 MHz, CDCl3): δ 8.32 (d, J = 5.5 Hz, 1H), 7.38 (s, 1H), 7.19 (d, J = 8.3 Hz, 1H), 6.80 (d, J = 8.3 Hz, 1H), 6.46 (t, J = 5.5 Hz, 1H), 4.82–4.74 (m, 2H), 4.65 (t, J = 8.8 Hz, 2H), 4.51 (dd, J = 12.7, 9.5 Hz, 2H), 3.24 (t, J = 8.8 Hz, 2H), 2.57 (d, J = 1.3 Hz, 3H), 1.53 (d, J = 1.7 Hz, 9H); HRMS m/z calcd for C23H25N5O3S 454.5450; found 452.6696 (M+H+).

#### 3.1.10. General Syntheses of Compounds 11a–d

*Tert*-Butyl *(S)*-1-(2-((1-(Cyclopropanecarbonyl)Piperidin-3-yl)Amino)Pyrimidin-4-yl)-2-(3,4-Dichlorophenyl)-4,6-Dihydropyrrolo[3,4-d]Imidazole-5(1*H*)-Carboxylate **(11a)**

Compound **9a** (0.16 mmol) was dissolved in 2 mL of methanol, and then potassium peroxomonosulfate (0.8 mmol) dissolved in 2 mL of water was added, followed by stirring at ambient temperature for 2 h. Methanol was concentrated and extracted with ethyl acetate and washed with water and brine, followed by drying with anhydrous magnesium sulfate and concentration of the solvent in vacuo to obtain compound **10a** as a white solid. Next, (S)-(3-aminopiperidin-1-yl)(cyclopropyl)methanone (0.32 mmol) was dissolved in 1 mL of dimethylformamide, and 44.5 µL (0.32 mmol) of triethylamine were added. Then, compound **10a** (0.14 mmol) dissolved in 3 mL of tetrahydrofuran was added and stirred at 80 °C for 24 h. Tetrahydrofuran was concentrated in vacuo, extracted with ethyl acetate, and washed with water and brine. After drying over anhydrous magnesium sulfate and concentration, the product was purified by column chromatography on silica gel using a mobile phase of EA:HEX (5:1) to obtain compound **11a** as a yellow solid. (48%); ^1^H NMR (400 MHz, CDCl3): δ 8.20 (s, 1H), 7.66 (s, 1H), 7.47 (d, J = 8.3 Hz, 1H), 7.35–7.27 (m, 1H), 6.13 (d, J = 5.4 Hz, 1H), 4.71 (d, J = 33.9 Hz, 2H), 4.49 (d, J = 25.9 Hz, 2H), 3.75 (d, J = 31.6 Hz, 5H), 1.79–1.59 (m, 5H), 1.52 (d, J = 2.8 Hz, 9H), 1.01 (s, 2H), 0.77 (s, 2H); HRMS m/z calcd for C29H33Cl2N7O3 598.5290; found 599.5036 (M+H+).

*Tert*-Butyl *(S)*-1-(2-((1-(Cyclopropanecarbonyl)Piperidin-3-yl)Amino)Pyrimidin-4-yl)-2-(Naphthalen-2-yl)-4,6-Dihydropyrrolo[3,4-d]Imidazole-5(1*H*)-Carboxylate **(11b)**

Compound **11b** was obtained as a yellow solid by the same procedure as above. (38%); ^1^H NMR (400 MHz, MeOD): δ 8.34–8.26 (m, 1H), 7.76–7.70 (m, 1H), 7.62 (dd, J = 8.3, 1.1 Hz, 1H), 7.41 (ddd, J = 8.2, 4.0, 2.0 Hz, 1H), 6.31 (d, J = 5.2 Hz, 1H), 4.77 (d, J = 12.9 Hz, 2H), 4.48 (d, J = 2.6 Hz, 2H), 3.96–3.54 (m, 4H), 2.32–2.03 (m, 4H), 1.56 (d, J = 2.5 Hz, 9H), 0.94–0.85 (m, 4H); HRMS m/z calcd for C33H37N7O3 579.2958; found 580.5351 (M+H).

*Tert*-Butyl *(S)*-2-(Benzo[d][1,3]Dioxol-5-yl)-1-(2-((1-(Cyclopropanecarbonyl)Piperidin-3-yl)Amino)Pyrimidin-4-yl)-4,6-Dihydropyrrolo[3,4-d]Imidazole-5(1*H*)-Carboxylate **(11c)**

Compound **11c** was obtained as a yellow solid by the same procedure as above. (52%); ^1^H NMR (400 MHz, CDCl3): δ 8.11 (s, 1H), 6.98–6.88 (m, 2H), 6.81 (d, J = 8.0 Hz, 1H), 6.02 (d, J = 8.7 Hz, 3H), 4.69 (d, J = 27.0 Hz, 2H), 4.46 (d, J = 26.1 Hz, 2H), 3.97–3.52 (m, 5H), 1.82–1.56 (m, 5H), 1.50 (d, J = 2.4 Hz, 9H), 1.02–0.92 (m, 2H), 0.76 (d, J = 3.2 Hz, 2H); HRMS m/z calcd for C30H35N7O5 573.6540; found 574.4855 (M+H).

*Tert*-Butyl *(S)*-1-(2-((1-(Cyclopropanecarbonyl)Piperidin-3-yl)Amino)Pyrimidin-4-yl)-2-(2,3-Dihydrobenzofuran-5-yl)-4,6-Dihydropyrrolo[3,4-d]Imidazole-5(1*H*)-Carboxylate **(11d)**

Compound **11d** was obtained as a yellow solid by the same procedure as above. (52%); ^1^H NMR (400 MHz, CDCl3): δ 8.09 (s, 1H), 7.35 (d, J = 8.2 Hz, 1H), 7.17 (s, 1H), 6.76 (d, J = 8.3 Hz, 1H), 6.07 (s, 1H), 4.71 (d, J = 29.1 Hz, 2H), 4.62 (t, J = 8.8 Hz, 2H), 4.47 (d, J = 26.7 Hz, 2H), 3.70 (dd, J = 91.3, 72.0 Hz, 6H), 3.22 (t, J = 8.7 Hz, 2H), 1.80–1.56 (m, 5H), 1.51 (d, J = 2.5 Hz, 9H), 1.00 (s, 2H), 0.88 (d, J = 6.5 Hz, 2H); HRMS m/z calcd for C31H37N7O4 571.6820; found 572.2907 (M+H).

#### 3.1.11. General Syntheses of Compounds 12a–d

*Tert*-Butyl *(R)*-1-(2-((1-(Cyclopropanecarbonyl)Pyrrolidin-3-yl)Amino)Pyrimidin-4-yl)-2-(3,4-Dichlorophenyl)-4,6-Dihydropyrrolo[3,4-d]Imidazole-5(1*H*)-Carboxylate **(12a)**

Compound **9a** (0.16 mmol) was dissolved in 2 mL of methanol, and then potassium peroxomonosulfate (0.8 mmol) dissolved in 2 mL of water was added, followed by stirring at ambient temperature for 2 h. Methanol was concentrated and extracted with ethyl acetate and washed with water and brine. This was followed by drying with anhydrous magnesium sulfate and concentration of the solvent in vacuo to obtain compound **10a** as white solid. Next, (R)-(3-aminopirrolidine-1-yl)(cyclopropyl)methanone (0.32 mmol) was dissolved in 1 mL of dimethylformamide, and 44.5 µl (0.32 mmol) of triethylamine were added. Then, compound **10a** (0.14 mmol) dissolved in 3 mL of tetrahydrofuran was added and stirred at 80 °C for 24 h. Tetrahydrofuran was concentrated in vacuo, extracted with ethyl acetate, and washed with water and brine. After drying over anhydrous magnesium sulfate and concentration, the product was purified by column chromatography on silica gel using a mobile phase of EA: HEX (5: 1) to obtain compound **12a** as a yellow solid. (38%); ^1^H NMR (400 MHz, MeOD): δ 8.34–8.26 (m, 1H), 7.76–7.70 (m, 1H), 7.62 (dd, J = 8.3, 1.1 Hz, 1H), 7.41 (ddd, J = 8.2, 4.0, 2.0 Hz, 1H), 6.31 (d, J = 5.2 Hz, 1H), 4.77 (d, J = 12.9 Hz, 2H), 4.48 (d, J = 2.6 Hz, 2H), 3.96–3.54 (m, 4H), 2.32–2.03 (m, 4H), 1.56 (d, J = 2.5 Hz, 9H), 0.94–0.85 (m, 4H); HRMS m/z calcd for C28H31Cl2N7O3 584.5020; found 585.3686 (M+H).

*Tert*-Butyl *(R)*-1-(2-((1-(Cyclopropanecarbonyl)Pyrrolidin-3-yl)Amino)Pyrimidin-4-yl)-2-(Naphthalen-2-yl)-4,6-Dihydropyrrolo[3,4-d]Imidazole-5(1*H*)-Carboxylate **(12b)**

Compound **12b** was obtained as a yellow solid by the same procedure as above. (30%); ^1^H NMR (400 MHz, CDCl3): δ 8.14–8.00 (m, 2H), 7.85 (d, J = 7.9 Hz, 3H), 7.58–7.49 (m, 3H), 6.16–6.03 (m, 1H), 4.77 (d, J = 16.4 Hz, 2H), 4.53 (d, J = 26.8 Hz, 2H), 3.79–3.50 (m, 4H), 2.18 (d, J = 13.6 Hz, 4H), 1.53 (s, 9H), 1.00 (s, 2H), 0.78 (d, J = 5.1 Hz, 2H); HRMS m/z calcd for C32H35N7O3 565.6780; found 566.2753 (M+H).

*Tert*-Butyl *(R)*-2-(Benzo[d][1,3]Dioxol-5-yl)-1-(2-((1-(Cyclopropanecarbonyl) Pyrrolidine-3-yl)Amino)Pyrimidin-4-yl)-4,6-Dihydropyrrolo[3,4-d]Imidazole-5(1*H*)-Carboxylate (12c)

Compound **12c** was obtained as a yellow solid by the same procedure as above. (44%); ^1^H NMR (400 MHz, CDCl3): δ 8.11 (dd, J = 11.3, 5.3 Hz, 1H), 6.98–6.92 (m, 2H), 6.81 (dd, J = 8.0, 5.6 Hz, 1H), 6.06–6.00 (m, 3H), 4.71 (dd, J = 23.3, 9.4 Hz, 2H), 4.43 (s, 2H), 3.80–3.58 (m, 4H), 2.37–1.95 (m, 4H), 1.52–1.48 (m, 9H), 0.98 (d, J = 4.8 Hz, 2H), 0.78–0.74 (m, 2H); HRMS m/z calcd for C29H33N7O5 559.6270; found 560.4778 (M+H).

*Tert*-Butyl *(R)*-1-(2-((1-(Cyclopropanecarbonyl)Pyrrolidin-3-yl)Amino)Pyrimidin-4-yl)-2-(2,3-Dihydrobenzofuran-5-yl)-4,6-Dihydropyrrolo[3,4-d]Imidazole-5(1*H*)-Carboxylate **(12d)**

Compound **12d** was obtained as a yellow solid by the same procedure as above. (48%); ^1^H NMR (400 MHz, CDCl3): δ 8.09 (dd, J = 12.1, 5.2 Hz, 1H), 7.35 (d, J = 10.3 Hz, 1H), 7.21–7.14 (m, 1H), 6.77 (dd, J = 8.2, 3.7 Hz, 1H), 6.10 (ddd, J = 15.8, 12.2, 5.4 Hz, 1H), 4.82–4.68 (m, 2H), 4.63 (d, J = 8.8 Hz, 2H), 4.43 (s, 2H), 3.99–3.53 (m, 5H), 3.22 (t, J = 8.7 Hz, 2H), 2.39–2.05 (m, 2H), 1.92 (dd, J = 12.5, 6.2 Hz, 1H), 1.53–1.48 (m, 9H), 0.77 (dd, J = 7.8, 4.7 Hz, 4H); HRMS m/z calcd for C30H35N7O4 557.6550; found 558.2751 (M+H).

#### 3.1.12. General Syntheses of Compounds 13a–d

*Tert*-Butyl *(S)*-1-(2-((1-(Cyclopropanecarbonyl)Pyrrolidin-3-yl)Amino)Pyrimidin-4-yl)-2-(3,4-Dichlorophenyl)-4,6-Dihydropyrrolo[3,4-d]Imidazole-5(1*H*)-Carboxylate **(13a)**

Compound **9a** (0.16 mmol) was dissolved in 2 mL of methanol, and then potassium peroxomonosulfate (0.8 mmol) dissolved in 2 mL of water was added, followed by stirring at ambient temperature for 2 h. Methanol was concentrated and extracted with ethyl acetate and washed with water and brine, followed by drying with anhydrous magnesium sulfate and concentration of the solvent in vacuo to obtain compound **10a** as white solid. (S)-(3-aminopirrolidine-1-yl)(cyclopropyl)methanone (0.32 mmol) was dissolved in 1 mL of dimethylformamide, and 44.5 µL (0.32 mmol) of triethylamine were added. Then, compound **10a** (0.14 mmol) dissolved in 3 mL of tetrahydrofuran was added and stirred at 80 °C for 24 h. Tetrahydrofuran was concentrated in vacuo, extracted with ethyl acetate, and washed with water and brine. After drying over anhydrous magnesium sulfate and concentration, the product was purified by column chromatography on silica gel using a mobile phase of EA: HEX (5: 1) to obtain compound **13a** as a yellow solid. (32%); ^1^H NMR (400 MHz, MeOD): δ 8.34–8.26 (m, 1H), 7.76–7.70 (m, 1H), 7.62 (dd, J = 8.3, 1.1 Hz, 1H), 7.41 (ddd, J = 8.2, 4.0, 2.0 Hz, 1H), 6.31 (d, J = 5.2 Hz, 1H), 4.77 (d, J = 12.9 Hz, 2H), 4.48 (d, J = 2.6 Hz, 2H), 3.89–3.48 (m, 4H), 2.25–2.02 (m, 4H), 1.56 (d, J = 2.5 Hz, 9H), 0.96–0.84 (m, 4H); HRMS m/z calcd for C28H31Cl2N7O3 584.5020; found 585.3209 (M+H).

*Tert*-Butyl *(S)*-1-(2-((1-(Cyclopropanecarbonyl)Pyrrolidin-3-yl)Amino)Pyrimidin-4-yl)-2-(Naphthalen-2-yl)-4,6-Dihydropyrrolo[3,4-d]Imidazole-5(1*H*)-Carboxylate **(13b)**

Compound **13b** was obtained as a yellow solid by the same procedure as above. (30%); ^1^H NMR (400 MHz, CDCl3): δ 8.10–8.02 (m, 2H), 7.85 (d, J = 7.2 Hz, 3H), 7.56–7.48 (m, 3H), 6.18–6.02 (m, 1H), 4.77 (d, J = 16.3 Hz, 2H), 4.53 (d, J = 27.3 Hz, 2H), 3.76–3.55 (m, 4H), 2.28–2.03 (m, 4H), 1.53 (s, 9H), 1.00–0.95 (m, 2H), 0.77 (d, J = 4.3 Hz, 2H); HRMS m/z calcd for C32H35N7O3 565.6780; found 566.5274 (M+H).

*Tert*-Butyl *(S)*-2-(Benzo[d][1,3]Dioxol-5-yl)-1-(2-((1-(Cyclopropanecarbonyl) Pyrrolidine-3-yl)Amino)Pyrimidin-4-yl)-4,6-Dihydropyrrolo[3,4-d]Imidazole-5(1*H*)-Carboxylate **(13c)**

Compound **13c** was obtained as a yellow solid by the same procedure as above. (26%); ^1^H NMR (400 MHz, CDCl3): δ 8.18–8.09 (m, 1H), 6.97 (ddd, J = 8.7, 5.3, 1.5 Hz, 2H), 6.83 (d, J = 8.0 Hz, 1H), 6.19–6.06 (m, 1H), 6.02 (d, J = 1.1 Hz, 2H), 4.83–4.64 (m, 2H), 4.57–4.42 (m, 2H), 4.03–3.52 (m, 5H), 2.44–2.07 (m, 2H), 1.99–1.81 (m, 1H), 1.52–1.43 (m, 9H), 1.01 (d, J = 2.4 Hz, 2H), 0.78 (dd, J = 7.5, 4.3 Hz, 2H); HRMS m/z calcd for C29H33N7O5 559.6270; found 560.2543 (M+H).

*Tert*-Butyl *(S)*-1-(2-((1-(Cyclopropanecarbonyl)Pyrrolidin-3-yl)Amino)Pyrimidin-4-yl)-2-(2,3-Dihydrobenzofuran-5-yl)-4,6-Dihydropyrrolo[3,4-d]Imidazole-5(1*H*)-Carboxylate **(13d)**

Compound **13d** was obtained as a yellow solid by the same procedure as above. (52%); ^1^H NMR (400 MHz, CDCl3) δ 8.14–8.03 (m, 1H), 7.35 (d, J = 11.0 Hz, 1H), 7.18 (d, J = 7.2 Hz, 1H), 6.78 (dd, J = 8.2, 3.9 Hz, 1H), 6.10 (ddd, J = 15.7, 12.5, 5.5 Hz, 1H), 4.75 (d, J = 1.9 Hz, 2H), 4.62 (d, J = 8.8 Hz, 2H), 4.44 (s, 2H), 3.85–3.53 (m, 5H), 3.21 (d, J = 8.7 Hz, 2H), 2.37–2.07 (m, 2H), 1.93 (dd, J = 12.4, 6.0 Hz, 1H), 1.54–1.49 (m, 9H), 1.00 (d, J = 2.3 Hz, 2H), 0.79–0.74 (m, 2H); HRMS m/z calcd for C30H35N7O4 557.6550; found 558.1865 (M+H).

#### 3.1.13. General Syntheses of Compounds 14a and 14c

*Tert*-Butyl *(S)*-1-(2-((1-(Cyclobutanecarbonyl)Piperidin-3-yl)Amino)Pyrimidin-4-yl)-2-(3,4-Dichlorophenyl)-4,6-Dihydropyrrolo[3,4-d]Imidazole-5(1*H*)-Carboxylate **(14a)**

Compound **9a** (0.16 mmol) was dissolved in 2 mL of methanol, and then potassium peroxomonosulfate (0.8 mmol) dissolved in 2 mL of water was added, followed by stirring at ambient temperature for 2 h. Methanol was concentrated and extracted with ethyl acetate and washed with water and brine, followed by drying with anhydrous magnesium sulfate and concentration of the solvent in vacuo to obtain compound **10a** as white solid. (S)-(3-aminopiperidin-1-yl)(cyclobutyl)methanone (0.32 mmol) was dissolved in 1 mL of dimethylformamide, and 44.5 µl (0.32 mmol) of triethylamine were added. Then, compound **10a** (0.14 mmol) dissolved in 3 mL of tetrahydrofuran was added and stirred at 80 °C for 24 h. Tetrahydrofuran was concentrated in vacuo, extracted with ethyl acetate, and washed with water and brine. After drying over anhydrous magnesium sulfate and concentration, the product was purified by column chromatography on silica gel using a mobile phase of EA: HEX (5: 1) to obtain compound **14a** as a yellow solid. (49%); ^1^H NMR (400 MHz, CDCl_3_): δ 8.25–8.05 (m, 1H), 7.71–7.62 (m, 1H), 7.47 (dd, J = 10.5, 4.8 Hz, 1H), 7.29 (d, J = 7.6 Hz, 1H), 6.16 (d, J = 5.0 Hz, 1H), 4.70 (dd, J = 31.8, 22.8 Hz, 2H), 4.46 (t, J = 17.4 Hz, 2H), 3.97–3.54 (m, 3H), 3.42–3.13 (m, 3H), 2.39–2.09 (m, 4H), 2.00–1.62 (m, 6H), 1.50 (d, J = 2.4 Hz, 9H); HRMS m/z calcd for C30H35Cl2N7O3 612.5560; found 613.3670 (M+H).

*Tert*-Butyl *(S)*-2-(Benzo[d][1,3]Dioxol-5-yl)-1-(2-((1-(Cyclobutanecarbonyl)Piperidin-3-yl)Amino)Pyrimidin-4-yl)-4,6-Dihydropyrrolo[3,4-d]Imidazole-5(1*H*)-Carboxylate **(14c)**

Compound **14c** was obtained as a yellow solid by the same procedure as above. (20%); ^1^H NMR (400 MHz, MeOD): δ 8.28–8.14 (m, 1H), 7.03–6.83 (m, 3H), 6.28 (s, 1H), 6.04 (d, J = 2.2 Hz, 2H), 4.80 (s, 2H), 4.47 (d, J = 12.3 Hz, 2H), 4.06 (d, J = 81.6 Hz, 2H), 3.12–3.02 (m, 1H), 2.94 (s, 1H), 2.15–1.43 (m, 12H); HRMS m/z calcd for C31H37N7O5 587.6810; found 588.5726 (M+H).

#### 3.1.14. General Syntheses of Tert-Butyl (S)-1-(2-((1-(Cyclopentanecarbonyl)Piperidin-3-yl)Amino)Pyrimidin-4-yl)-2-(3,4-Dichlorophenyl)-4,6-Dihydropyrrolo[3,4-d]Imidazole-5(1H)-Carboxylate (15a)

Compound **9a** (0.16 mmol) was dissolved in 2 mL of methanol, and then potassium peroxomonosulfate (0.8 mmol) dissolved in 2 mL of water was added, followed by stirring at ambient temperature for 2 h. Methanol was concentrated and extracted with ethyl acetate and washed with water and brine, followed by drying with anhydrous magnesium sulfate and concentration of the solvent in vacuo to obtain compound **10a** as white solid. (S)-(3-aminopiperidin-1-yl)(cyclopentyl)methanone (0.32 mmol) was dissolved in 1 mL of dimethylformamide, and 44.5 µl (0.32 mmol) of triethylamine were added. Then, compound **10a** (0.14 mmol) dissolved in 3 mL of tetrahydrofuran was added and stirred at 80 °C for 24 h. Tetrahydrofuran was concentrated in *vacuo*, extracted with ethyl acetate, and washed with water and brine. After drying over anhydrous magnesium sulfate and concentration, the product was purified by column chromatography on silica gel using a mobile phase of EA: HEX (5: 1) to obtain compound **15a** as a yellow solid. (8%); ^1^H NMR (400 MHz, CDCl_3_): δ 8.19 (t, J = 30.4 Hz, 1H), 7.68 (s, 1H), 7.51 (dd, J = 13.1, 6.2 Hz, 1H), 7.35–7.28 (m, 1H), 6.22 (s, 1H), 4.85–4.66 (m, 2H), 4.49 (d, J = 20.4 Hz, 2H), 3.80–3.50 (m, 4H), 2.92 (s, 2H), 1.75 (d, J = 38.6 Hz, 12H), 1.51 (d, J = 2.5 Hz, 9H); HRMS m/z calcd for C31H37Cl2N7O3 626.5830; found 627.3383 (M+H).

#### 3.1.15. General Syntheses of Compounds 17a–d, 18a–d, 19a–d, 20a, 20c, and 21a

*(S)*-1-(2-((1-(Cyclopropanecarbonyl)Piperidin-3-yl)Amino)Pyrimidin-4-yl)-2-(3,4-Dichlorophenyl)-4,6-Dihydropyrrolo[3,4-d]Imidazole-5(1*H*)-Carboxamide **(17a)**

Compound **11a** (0.05 mol) was dissolved in 0.5 mL of 1,4-dioxane, and 0.25 mL of 4 M HCl in 1,4-dioxane was added. The mixture was stirred at ambient temperature for 1 h 30 min. After concentrating 1,4-dioxane in vacuo, compound **16** was obtained. Compound **16** (0.048 mol) was dissolved in 0.48 mL of 1,4-dioxane, and then phenyl carbamate (0.048 mmol) and triethylamine (0.048 mmol) were added, and the mixture was stirred at ambient temperature for 24 h. 1,4-Dioxane was concentrated in vacuo, extracted with ethyl acetate, and washed with water and brine. After drying with anhydrous magnesium sulfate and concentration of the solvent in vacuo, the product was purified by column chromatography on silica gel using a mobile phase of MC:MeOH (10:1) to obtain compound **17a** as a white solid. (31%); ^1^H NMR (400 MHz, MeOD): δ 8.36–8.23 (m, 1H), 7.69 (s, 1H), 7.59 (d, J = 8.2 Hz, 1H), 7.37 (t, J = 6.3 Hz, 1H), 6.33 (d, J = 75.5 Hz, 1H), 4.76 (s, 2H), 4.49 (dd, J = 11.6, 8.5 Hz, 2H), 4.06 (d, J = 60.6 Hz, 2H), 3.40–3.05 (m, 5H), 2.10–1.40 (m, 5H), 0.96–0.53 (m, 4H); HRMS m/z calcd for C25H26Cl2N8O2 541.4370; found 542.4286 (M+H).

*(S)*-1-(2-((1-(Cyclopropanecarbonyl)piperidin-3-yl)Amino)Pyrimidin-4-yl)-2-(Naphthalen-2-yl)-4,6-Dihydropyrrolo[3,4-d]Imidazole-5(1*H*)-Carboxamide **(17b)**

Compound **17b** was obtained as a white solid by the same procedure as above. (31%); ^1^H NMR (400 MHz, MeOD): δ 8.29–8.05 (m, 2H), 7.92 (s, 3H), 7.65–7.39 (m, 3H), 6.26 (d, J = 102.0 Hz, 1H), 4.69 (d, J = 17.1 Hz, 2H), 4.63–4.48 (m, 2H), 4.19–3.72 (m, 2H), 3.29–2.31 (m, 3H), 2.17–1.43 (m, 5H), 0.90 (dt, J = 73.7, 30.3 Hz, 4H); HRMS m/z calcd for C29H30N8O2 522.6130; found 523.4595 (M+H).

*(S)*-2-(Benzo[d][1,3]Dioxol-5-yl)-1-(2-((1-(Cyclopropanecarbonyl)Piperidin-3-yl)Amino)Pyrimidin-4-yl)-4,6-Dihydropyrrolo[3,4-d]Imidazole-5(1*H*)-Carboxamide **(17c)**

Compound **17c** was obtained as a white solid by the same procedure as above. (47%); ^1^H NMR (400 MHz, MeOD): δ 8.28–8.11 (m, 1H), 6.92 (dt, J = 20.5, 4.7 Hz, 3H), 6.34–5.99 (m, 3H), 4.76 (s, 2H), 4.47 (s, 2H), 3.98 (ddd, J = 139.3, 60.2, 35.9 Hz, 3H), 2.14–1.45 (m, 7H), 0.92–0.68 (m, 4H); HRMS m/z calcd for C26H28N8O4 516.5620; found 517.4097 (M+H).

*(S)*-1-(2-((1-(Cyclopropanecarbonyl)Piperidin-3-yl)Amino)Pyrimidin-4-yl)-2-(2,3-Dihydrobenzofuran-5-yl)-4,6-Dihydropyrrolo[3,4-d]Imidazole-5(1*H*)-Carboxamide **(17d)**

Compound **17d** was obtained as a white solid by the same procedure as above. (40%); ^1^H NMR (400 MHz, MeOD): δ 8.24–8.09 (m, 1H), 7.32 (s, 1H), 7.19 (d, J = 7.8 Hz, 1H), 6.78 (d, J = 8.1 Hz, 1H), 6.17 (d, J = 77.2 Hz, 1H), 4.77 (s, 2H), 4.61 (t, J = 8.8 Hz, 2H), 4.48 (d, J = 2.8 Hz, 2H), 4.26–3.52 (m, 3H), 3.23 (dd, J = 10.2, 8.0 Hz, 2H), 2.19–1.43 (m, 6H), 1.31 (dd, J = 12.7, 5.3 Hz, 1H), 0.81 (ddd, J = 94.9, 52.5, 36.9 Hz, 4H); HRMS m/z calcd for C27H30N8O3 514.5900; found 515.4652 (M+H).

*(R)*-1-(2-((1-(Cyclopropanecarbonyl)Pyrrolidin-3-yl)Amino)Pyrimidin-4-yl)-2-(3,4-Dichlorophenyl)-4,6-Dihydropyrrolo[3,4-d]Imidazole-5(1*H*)-Carboxamide **(18a)**

Compound **18a** was obtained as a white solid by the same procedure as above. (21%); ^1^H NMR (400 MHz, MeOD): δ 8.29 (dd, J = 10.5, 5.3 Hz, 1H), 7.72 (t, J = 1.8 Hz, 1H), 7.61 (dd, J = 8.3, 2.0 Hz, 1H), 7.42–7.38 (m, 1H), 6.30 (s, 1H), 4.78 (d, J = 3.0 Hz, 2H), 4.50 (s, 2H), 3.90–3.42 (m, 4H), 2.30–1.71 (m, 4H), 0.87 (ddd, J = 10.2, 7.3, 3.4 Hz, 4H); HRMS m/z calcd for C24H24Cl2N8O2 527.4100; found 528.3486 (M+H).

*(R)*-1-(2-((1-(Cyclopropanecarbonyl)Pyrrolidin-3-yl)Amino)Pyrimidin-4-yl)-2-(Naphthalen-2-yl)-4,6-Dihydropyrrolo[3,4-d]Imidazole-5(1*H*)-Carboxamide **(18b)**

Compound **18b** was obtained as a white solid by the same procedure as above. (37%); ^1^H NMR (400 MHz, MeOD): δ 8.26–8.12 (m, 1H), 8.06 (d, J = 3.9 Hz, 1H), 7.96–7.85 (m, 3H), 7.62–7.46 (m, 3H), 6.24 (s, 1H), 4.82 (d, J = 2.4 Hz, 2H), 4.53 (t, J = 3.0 Hz, 2H), 3.99–3.41 (m, 4H), 1.86 (dt, J = 102.9, 33.3 Hz, 4H), 0.96–0.70 (m, 4H); HRMS m/z calcd for C28H28N8O2 508.5860; found 509.4154 (M+H).

*(R)*-2-(Benzo[d][1,3]Dioxol-5-yl)-1-(2-((1-(Cyclopropanecarbonyl)Pyrrolidin-3-yl)Amino)Pyrimidin-4-yl)-4,6-Dihydropyrrolo[3,4-d]Imidazole-5(1*H*)-Carboxamide **(18c)**

Compound **18c** was obtained as a white solid by the same procedure as above. (13%); ^1^H NMR (400 MHz, MeOD): δ 8.21 (dd, J = 11.0, 5.4 Hz, 1H), 6.97 (tt, J = 3.1, 1.4 Hz, 2H), 6.90 (d, J = 8.5 Hz, 1H), 6.18 (s, 1H), 6.04 (dd, J = 2.5, 1.3 Hz, 2H), 4.78 (s, 2H), 4.49 (d, J = 3.0 Hz, 2H), 3.93–3.39 (m, 5H), 2.40–1.73 (m, 3H), 0.87 (dddd, J = 12.5, 9.8, 5.8, 2.2 Hz, 4H); HRMS m/z calcd for C25H26N8O4 502.5350; found 503.4016 (M+H).

*(R)*-1-(2-((1-(Cyclopropanecarbonyl)Pyrrolidin-3-yl)Amino)Pyrimidin-4-yl)-2-(2,3-Dihydrobenzofuran-5-yl)-4,6-Dihydropyrrolo[3,4-d]Imidazole-5(1*H*)-Carboxamide **(18d)**

Compound **18d** was obtained as a white solid by the same procedure as above. (40%); ^1^H NMR (400 MHz, MeOD): δ 8.17 (dd, J = 12.3, 5.4 Hz, 1H), 7.33 (dd, J = 5.0, 1.3 Hz, 1H), 7.19 (d, J = 8.3 Hz, 1H), 6.79 (dd, J = 8.3, 1.6 Hz, 1H), 6.13 (s, 1H), 4.77 (s, 2H), 4.61 (td, J = 8.8, 2.0 Hz, 2H), 4.48 (d, J = 2.8 Hz, 2H), 4.37 (s, 1H), 3.83 (dddd, J = 29.1, 23.2, 18.4, 16.3 Hz, 2H), 3.68–3.38 (m, 2H), 3.23 (t, J = 8.7 Hz, 2H), 2.36–1.95 (m, 2H), 1.87–1.71 (m, 1H), 0.97–0.76 (m, 4H); HRMS m/z calcd for C26H28N8O3 500.5630; found 501.4210 (M+H).

*(S*)-1-(2-((1-(Cyclopropanecarbonyl)Pyrrolidin-3-yl)Amino)Pyrimidin-4-yl)-2-(3,4-Dichlorophenyl)-4,6-Dihydropyrrolo[3,4-d]Imidazole-5(1*H*)-Carboxamide **(19a)**

Compound **19a** was obtained as a white solid by the same procedure as above. (35%); ^1^H NMR (400 MHz, MeOD): δ 8.29 (dd, J = 10.6, 5.3 Hz, 1H), 7.72 (t, J = 1.8 Hz, 1H), 7.61 (dd, J = 8.3, 2.0 Hz, 1H), 7.42–7.37 (m, 1H), 6.31 (s, 1H), 4.77 (d, J = 2.9 Hz, 2H), 4.50 (s, 2H), 3.92–3.46 (m, 4H), 2.30–1.68 (m, 4H), 0.93–0.81 (m, 4H); HRMS m/z calcd for C24H24Cl2N8O2 527.4100; found 528.3846 (M+H).

*(S)*-1-(2-((1-(Cyclopropanecarbonyl)Pyrrolidin-3-yl)Amino)Pyrimidin-4-yl)-2-(3,4-Dichlorophenyl)-4,6-Dihydropyrrolo[3,4-d]Imidazole-5(1*H*)-Carboxamide **(19b)**

Compound **19b** was obtained as a white solid by the same procedure as above. (27%); ^1^H NMR (400 MHz, MeOD): δ 8.25–8.13 (m, 1H), 8.09–8.04 (m, 1H), 7.97–7.86 (m, 3H), 7.64–7.47 (m, 3H), 6.24 (s, 1H), 4.82 (d, J = 2.4 Hz, 2H), 4.53 (t, J = 2.9 Hz, 2H), 3.91–3.40 (m, 4H), 2.14–1.50 (m, 4H), 0.93–0.76 (m, 4H); HRMS m/z calcd for C28H28N8O2 508.5860; found 509.4514 (M+H).

*(S)*-2-(Benzo[d][1,3]Dioxol-5-yl)-1-(2-((1-(Cyclopropanecarbonyl)Pyrrolidin-3-yl)Amino)Pyrimidin-4-yl)-4,6-Dihydropyrrolo[3,4-d]Imidazole-5(1*H*)-Carboxamide **(19c)**

Compound **19c** was obtained as a white solid by the same procedure as above. (34%); ^1^H NMR (400 MHz, MeOD): δ 8.21 (dd, J = 11.0, 5.4 Hz, 1H), 7.01–6.95 (m, 2H), 6.90 (d, J = 8.5 Hz, 1H), 6.21 (d, J = 29.0 Hz, 1H), 6.07–6.02 (m, 2H), 4.78 (s, 2H), 4.48 (t, J = 3.0 Hz, 2H), 4.35 (s, 1H), 4.02–3.73 (m, 2H), 3.61–3.41 (m, 2H), 2.36–2.05 (m, 2H), 1.80 (dddd, J = 28.7, 12.7, 7.9, 4.7 Hz, 1H), 0.92–0.77 (m, 4H); HRMS m/z calcd for C25H26N8O4 502.5350; found 503.4160 (M+H).

*(S)*-1-(2-((1-(Cyclopropanecarbonyl)Pyrrolidin-3-yl)Amino)Pyrimidin-4-yl)-2-(2,3-Dihydrobenzofuran-5-yl)-4,6-Dihydropyrrolo[3,4-d]Imidazole-5(1*H*)-Carboxamide **(19d)**

Compound **19d** was obtained as a white solid by the same procedure as above. (30%); ^1^H NMR (400 MHz, MeOD): δ 8.18 (dd, J = 12.2, 5.4 Hz, 1H), 7.34 (d, J = 3.8 Hz, 1H), 7.20 (d, J = 8.3 Hz, 1H), 6.79 (dd, J = 8.3, 1.6 Hz, 1H), 6.13 (s, 1H), 4.78 (s, 2H), 4.61 (td, J = 8.8, 2.0 Hz, 2H), 4.48 (s, 2H), 4.38 (s, 1H), 4.07–3.69 (m, 2H), 3.65–3.38 (m, 2H), 3.23 (t, J = 8.7 Hz, 2H), 2.36–1.95 (m, 2H), 1.79 (dddd, J = 29.6, 12.7, 7.9, 4.7 Hz, 1H), 0.96–0.77 (m, 4H); HRMS m/z calcd for C26H28N8O3 500.5630; found 501.2041 (M+H).

*(S)*-1-(2-((1-(Cyclobutanecarbonyl)Piperidin-3-yl)Amino)Pyrimidin-4-yl)-2-(3,4-Dichlorophenyl)-4,6-Dihydropyrrolo[3,4-d]Imidazole-5(1*H*)-Carboxamide **(20a)**

Compound **20a** was obtained as a white solid by the same procedure as above. (24%); ^1^H NMR (400 MHz, MeOD): δ 8.40–8.22 (m, 1H), 7.71 (d, J = 15.5 Hz, 1H), 7.61 (dd, J = 8.3, 4.8 Hz, 1H), 7.43–7.35 (m, 1H), 6.39 (d, J = 97.2 Hz, 1H), 4.81 (s, 2H), 4.50 (dd, J = 12.3, 2.9 Hz, 2H), 3.68 (s, 1H), 3.53–3.39 (m, 1H), 3.03 (d, J = 16.4 Hz, 1H), 2.91 (dd, J = 18.5, 8.5 Hz, 1H), 2.42–2.17 (m, 4H), 2.12–1.98 (m, 2H), 1.91–1.71 (m, 3H), 1.66–1.41 (m, 3H).; HRMS m/z calcd for C26H28Cl2N8O2 555.4640; found 556.4531 (M+H).

*(S)*-2-(Benzo[d][1,3]Dioxol-5-yl)-1-(2-((1-(Cyclobutanecarbonyl)Piperidin-3-yl)Amino)Pyrimidin-4-yl)-4,6-Dihydropyrrolo[3,4-d]Imidazole-5(1*H*)-Carboxamide **(20c)**

Compound **20c** was obtained as a white solid by the same procedure as above. (29%); ^1^H NMR (400 MHz, MeOD): δ 8.28–8.14 (m, 1H), 7.03–6.83 (m, 3H), 6.20 (d, J = 61.5 Hz, 3H), 4.80 (s, 2H), 4.48 (s, 2H), 4.06 (d, J = 81.6 Hz, 2H), 3.13–3.02 (m, 1H), 2.94 (s, 1H), 2.15–1.43 (m, 12H); HRMS m/z calcd for C27H30N8O4 530.5890; found 531.2490 (M+H).

*(S)*-1-(2-((1-(Cyclopentanecarbonyl)Piperidin-3-yl)Amino)Pyrimidin-4-yl)-2-(3,4-Dichlorophenyl)-4,6-Dihydropyrrolo[3,4-d]Imidazole-5(1*H*)-Carboxamide **(21a)**

Compound **21a** was obtained as a white solid by the same procedure as above. (68%); ^1^H NMR (400 MHz, MeOD): δ 8.38–8.24 (m, 1H), 7.71 (d, J = 10.9 Hz, 1H), 7.60 (d, J = 8.3 Hz, 1H), 7.42–7.34 (m, 1H), 6.36 (d, J = 86.1 Hz, 1H), 4.82 (s, 2H), 4.52 (d, J = 9.3 Hz, 2H), 4.21 (s, 1H), 3.97 (d, J = 35.3 Hz, 1H), 3.26–3.04 (m, 2H), 2.87 (s, 2H), 2.06–1.56 (m, 12H); HRMS m/z calcd for C27H30Cl2N8O2 569.4910; found 570.3722 (M+H).

#### 3.1.16. General Synthesis of (*S*)-(1-(2-((1-(Cyclopropanecarbonyl)piperidin-3-yl)amino)pyrimidin-4-yl)-2-(3,4-dichlorophenyl)-4,6-dihydropyrrolo[3,4-d]Imidazol-5(1*H*)-yl)(4-hydroxypiperidin-1-yl)methanone (22a)

Compound **11a** (0.05 mol) was dissolved in 0.5 mL of 1,4-dioxane, and 0.25 mL of 4 M HCl in 1,4-dioxane was added. The mixture was stirred at ambient temperature for 1 h 30 min. After concentrating 1,4-dioxane in vacuo, compound **16** was obtained. Compound **16** (0.044 mmol) was dissolved in 0.44 mL of 1,4-dioxane and treated with 4-nitrophenyl chloroformate (0.044 mmol) and dimethylformamide (0.22 mL) and stirred at ambient temperature for 1 h. 4-Piperidinol (0.22 mmol) was added and stirred at ambient temperature for 48 h. We concentrated the 1,4-dioxane in vacuo, extracted with ethyl acetate, and washed with water and brine. Drying over anhydrous magnesium sulfate, the solvent was concentrated in vacuo and the product purified using column chromatography on silica gel using a mobile phase of MC: MeOH (10: 1) to afford compound **22a** as a white solid. (16%); ^1^H NMR (400 MHz, MeOD): δ 8.27 (dd, J = 17.3, 5.0 Hz, 1H), 7.70 (d, J = 1.9 Hz, 1H), 7.60 (d, J = 8.2 Hz, 1H), 7.38 (s, 1H), 6.29 (s, 1H), 4.95 (s, 2H), 4.58 (t, J = 11.4 Hz, 2H), 4.35 (s, 1H), 4.10 (dd, J = 14.3, 7.1 Hz, 1H), 3.82 (ddd, J = 12.9, 8.6, 3.9 Hz, 1H), 3.71 (d, J = 13.7 Hz, 2H), 3.45 (d, J = 28.8 Hz, 1H), 3.00 (dd, J = 66.0, 53.2 Hz, 4H), 1.90 (dd, J = 42.4, 32.4 Hz, 5H), 1.55 (td, J = 12.9, 3.6 Hz, 4H), 0.83 (dd, J = 32.6, 25.9 Hz, 4H); HRMS m/z calcd for C30H34Cl2N8O3 625.5550; found 626.4741 (M+H).

### 3.2. Molecular Modeling

Compounds were docked into the JNK3 structure (PDB: 3OY1). Protein and ligand preparations were performed with the Schrödinger Maestro program (New York, NY, USA) with standard settings, and Glide was used for docking and scoring. The 3D X-ray protein structures of JNK3 as a complex with a ligand were obtained from the PDB (code: 3OY1) and prepared using the Protein Preparation Wizard of the Schrödinger Maestro program. All water molecules were removed from the structure, and it was selected as a template. The structures of inhibitors were drawn using ChemDraw (Seoul, Korea), and their 3D conformations were generated using the Schrödinger LigPrep program with the OPLS 2005 force field (New York, NY, USA). Molecular dockings of compounds into the structure of JNK3 (PDB code: 3OY1) were carried out using Schrodinger Glide (Version 12.2).

### 3.3. Evaluation of IC_50_ Values and Selected Kinase Profiling

We used Reaction Biology Corp.’s Kinase HotSpot^SM^ service (www.reactionbiology.com) for IC_50_ determination of all compounds and kinase profiles. Assay protocol: in a final reaction volume of 25 μL, ATF, 5 μM, ATP 10 μM, and JNK3 (h) (5–10 mU) were incubated with 25 mM Tris (pH 7.5), 0.02 mM EGTA, 0.66 mg/mL myelin basic protein, 10 mM Mg acetate, and [γ-33P-ATP] (specific activity approx. 500 cpm/pmol, concentration as required). The reaction was initiated by the addition of the Mg-ATP mix. After incubation for 40 min at room temperature, the reaction was stopped by the addition of 5 μL of a 3% phosphoric acid solution. Then, 10 μL of the reaction was spotted onto a P30 filtermat and washed three times for 5 min in 75 mM phosphoric acid and once in methanol prior to drying and scintillation counting.

## 4. Conclusions

We successfully synthesized 1-pyrimidinyl-2-aryl-4,6-dihydropyrrolo[3,4-d]imidazole-5(1*H*)-carboxamide derivatives that were designed as novel JNK3 inhibitors with better inhibitory activity than the previous lead in an effort to maintain the main interactions identified through the previous docking study [14]. Sixteen compounds were synthesized and measured for their enzyme activity against JNK3. In particular, compounds **17a, 18a**, **17b,** and **18b** showed competitive activities against JNK3 with IC_50_ values of 10.4 nM, 2.69 nM, 4.81 nM, and 4.52 nM, respectively. Compound **18a** was, indeed, a selective JNK3 inhibitor with an excellent selectivity profile, especially compared to its activity against similar protein kinases such as p38α, Erk, and JNK1. We believe that this novel scaffold of 1-pyrimidinyl-2-aryl-4,6-dihydropyrrolo[3,4-d]imidazole-5(1*H*)-carboxamide derivatives will be highly useful in the development of JNK3-selective inhibitors as therapeutic agents for neurodegenerative diseases.

## Figures and Tables

**Table 1:** Enzymatic activities of 1-pyrimidinyl-2-aryl-4, 6-dihydropyrrolo[3,4-d]imidazole-5(1*H*)-carboxamide derivatives.

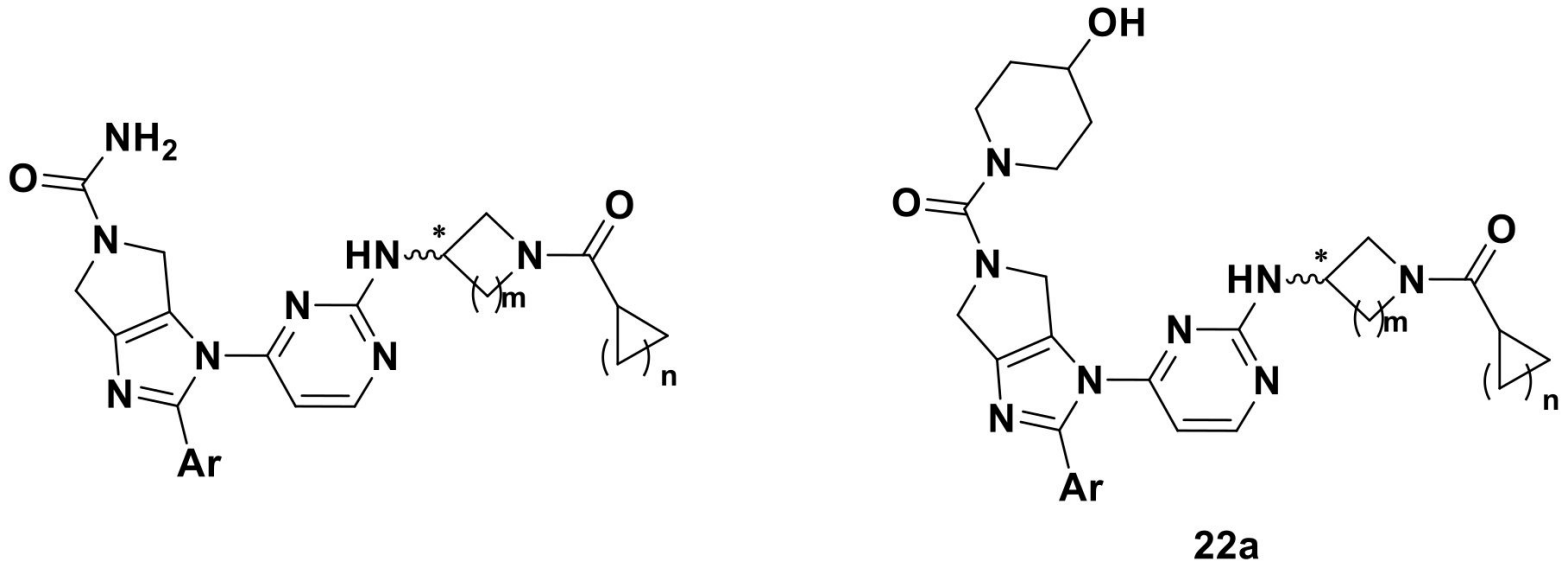					
No	Ar	m	n	* (R/S)	JNK3 IC_50_ (nM)
**17a**	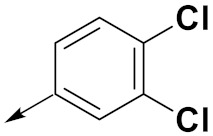	3	1	*S*	10.4
**18a**		2	1	*R*	2.69
**19a**		2	1	*S*	113
**20a**		3	2	*S*	29.7
**21a**		3	3	*S*	24.8
**22a**		3	1	*S*	18.6
**17b**	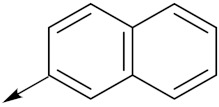	3	1	*S*	4.81
**18b**		2	1	*R*	4.52
**19b**		2	1	*S*	48.2
**17c**	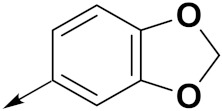	3	1	S	131
**18c**		2	1	R	37.3
**19c**		2	1	S	744
**20c**		3	2	S	225
**17d**	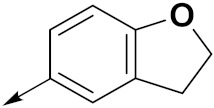	3	1	*S*	41.2
**18d**		2	1	*R*	16.7
**19d**		2	1	*S*	529
JNKI VIII [16,17]				5	

*: Stereocenter

**Table 2:** Percentages of enzymatic inhibition exerted by 10 μM of **18a** on 38 selected protein kinases [24] and enzymatic activities on selected protein kinases.

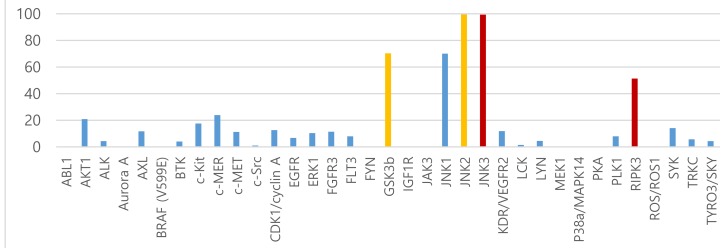			
Kinase	IC_50_ (M)		Selectivity/JNK3 of 18a
	18a	Control	
JNK3	2.69 × 10^−^^9^	5.00 × 10^−^^9^ (JNKI VIII)	-
GSK3β	5.53 × 10^−^^6^	2.30 × 10^−^^9^ (Staurosporine)	>2000 fold
JNK2	2.25 × 10^−^^7^	1.92 × 10^−^^6^ (Staurosporine)	>80 fold
RIPK3	>1.00 × 10^−^^5^	2.65 × 10^−^^7^ (GW5074)	>100 fold
